# Phenolic Compounds, Antioxidant Activities, and Inhibitory Effects on Digestive Enzymes of Different Cultivars of Okra (*Abelmoschus esculentus*)

**DOI:** 10.3390/molecules25061276

**Published:** 2020-03-11

**Authors:** Ding-Tao Wu, Xi-Rui Nie, Dan-Dan Shen, Hong-Yi Li, Li Zhao, Qing Zhang, De-Rong Lin, Wen Qin

**Affiliations:** College of Food Science, Sichuan Agricultural University, Ya’an 625014, China; niexirachel@163.com (X.-R.N.); dandanshensicau@163.com (D.-D.S.); Lhongyi45@163.com (H.-Y.L.); zhaoli0608@126.com (L.Z.); zhangqing@sicau.edu.cn (Q.Z.); linderong123@aliyun.com (D.-R.L.)

**Keywords:** okra, phenolic compound, HPLC analysis, antioxidant activity, digestive enzyme inhibition

## Abstract

In this study, the phenolic profiles and bioactivities of five representative cultivars of okra collected in China were investigated. Noticeable variations of phenolic compounds and their bioactivities were observed among these different cultivars of okra. The contents of total flavonoids (TFC) in “Shuiguo”, “Kalong 8”, “Kalong 3”, “Wufu”, and “Royal red” ranged from 1.75 to 3.39 mg RE/g DW, of which “Shuiguo” showed the highest TFC. Moreover, five individual phenolic compounds were found in okra by high performance liquid chromatography analysis, including isoquercitrin, protocatechuic acid, quercetin-3-O-gentiobioside, quercetin, and rutin, while isoquercitrin and quercetin-3-O-gentiobioside were detected as the main phenolic compounds in okra. Moreover, all tested okra exhibited significant antioxidant activities (2,2-diphenyl-1-picrylhydrazyl radical scavenging capacity, 2,2’-azino-bis (3-ethylenzthiazoline-6-sulphonic acid) radical scavenging capacity, and ferric reducing antioxidant power) and inhibitory effects on digestive enzymes (lipase, α-glucosidase, and α-amylase). Indeed, “Shuiguo” exhibited much better antioxidant activities and inhibitory activities on digestive enzymes, which might be attributed to its high TFC. Results suggested that okra, especially “Shuiguo”, could be developed as natural antioxidants and inhibitors against hyperlipidemia and hyperglycemia in the fields of functional foods and pharmaceuticals, which could meet the increasing demand for high-quality okra with health-promoting properties in China.

## 1. Introduction

The fruit of okra (*Abelmoschus esculentus* L. Moench), native to Africa, has been cultivated and used as food and folk medicine around the world due to its health-promoting benefits [1,2]. Various cultivars of okra fruits have also been cultivated widely in China, including *A. esculentus* cv. Wuxing, *A. esculentus* cv. Kalong 3, *A. esculentus* cv. Kalong 8, *A. esculentus* cv. Wufu, *A. esculentus* cv. Royal red, and *A. esculentus* cv. Shuiguo [3]. It is commonly considered that okra fruits can prevent diabetes and obesity [2,4]. In addition, it is also believed that okra fruits possess various bioactivities, such as anti-hyperlipidemic [1], antioxidant [5,6], anti-hyperglycemic [7], and neuroprotective activities [8]. Generally, polysaccharides and phenolic compounds are referred to the major bioactive components in okra fruits, which are also the sources of its various biological activities [9].

Generally, polysaccharides and their bioactivities are influenced by different cultivars of okra fruits whether in China or abroad [3,10]. Previous study has reported that the content of phenolics and flavonoids are significantly different in different cultivars of okra collected in Greece, and their antioxidant activities may be also influenced by different cultivars [11]. At present, growing evidence has documented that the content of phenolics can directly influence the digestive enzymes, which participate in the hydrolyzation of fats and carbohydrates in our daily diet [12]. However, the determination and comparison of phenolic compounds in different cultivars of okra cultivated in China and the correlations among phenolic compounds, antioxidant activity, anti-hyperlipidemic activity, and anti-hyperglycemic activity have seldom been investigated. Furthermore, qualitative and quantitative analysis of phenolic compounds in different cultivars of okra fruits is also important and necessary for the evaluation of their biological characteristics [13,14]. Thus, it is necessary to evaluate and compare the phenolic compounds and their bioactivities of different cultivars of okra fruits collected in China, so as to meet the increasing demand for high-quality vegetables with health-promoting properties in China.

In this study, in order to properly understand the phenolic compounds and their bioactivities of different cultivars of okra fruits collected in China, the phenolic profiles, antioxidant capacities, and inhibitory effects on digestive enzymes of five representative cultivars of okra fruits collected in China, including “Kalong 3”, “Kalong 8”, “Shuiguo”, “Wufu”, and “Royal red”, were systematically evaluated and compared.

## 2. Results and Discussion

### 2.1. Phenolic Compounds in Different Cultivars of Okra Fruits

Phenolic compounds are considered as one of the major bioactive components in okra fruits [2,5]. Therefore, phenolic compounds in different cultivars of okra fruits cultivated in China were investigated. The contents of total flavonoids (TFC) of the five representative okra fruits collected in China were determined and presented in Table 1. Significantly different levels (*p* < 0.05) of TFC were detected in “Shuiguo” (3.39 mg RE/g DW), compared to “Kalong 3” (3.22 mg RE/g DW), “Kalong 8” (3.03 mg RE/g DW), “Wufu” (2.94 mg RE/g DW), and “Royal red” (1.75 mg RE/g DW). Results showed that the TFC changed significantly among the five okra fruits, which was similar to previous studies [15,16]. In fact, the phenolic profiles of plants are directly affected by extrinsic and intrinsic factors, such as cultivar, maturity, and environmental conditions [17]. The interaction of these factors will influence the metabolism of plants, and then lead to produce different bioactive compounds, such as different type of phenolic compounds [15].

**Figure 1:**
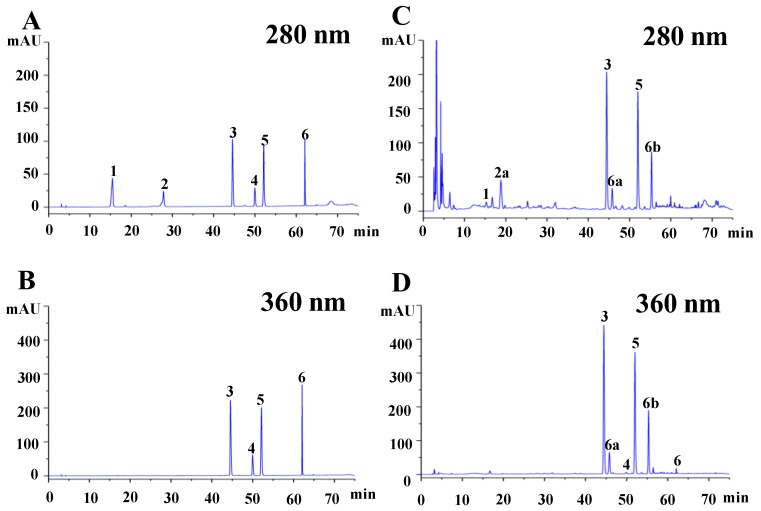
High performance liquid chromatograms of mixed standards (**A** and **B**) and the representative cultivar (“Shuiguo”) of okra fruit (**C** and **D**); **1**, protocatechuic acid; **2**, catechin; **3**, quercetin-3-O-gentiobioside; **4**, rutin; **5**, isoquercitrin; **6**, quercetin; **2a**, catechin derivative; **6a**, quercetin derivative a; **6b**, quercetin derivative b.

Thus, a total of six phenolic compounds were investigated in the okra fruit based on previous studies, including catechin, isoquercitrin, protocatechuic acid, quercetin, quercetin-3-O-gentiobioside, and rutin [2,15,18]. Figure 1A and Figure 1B showed the high-performance liquid chromatography (HPLC) chromatograms of the six mixed standards, and Figure 1C and Figure 1D showed the individual phenolic compounds in the representative cultivar (“Shuiguo”) of okra fruit. Results showed that five phenolic compounds, including isoquercitrin (UV λ_max_, 245 nm and 355 nm), protocatechuic acid (UV λ_max_, 260 nm and 293 nm), quercetin (UV λ_max_, 255 nm and 365 nm), quercetin-3-O-gentiobioside (UV λ_max_, 203 nm, 255 nm and 355 nm), and rutin (UV λ_max_, 212 nm, 257 nm and 354 nm) were detected in all the five okra fruits according to the HPLC retention time and UV spectra. Additionally, one catechin derivative and two quercetin derivatives might exist in all these five okra fruits according to their UV spectral information, which the UV spectra of catechin derivative and quercetin derivatives were the same to catechin (UV λ_max_, 225 nm and 280 nm) and quercetin (UV λ_max_, 255 nm and 365 nm), respectively.

The phenolic compounds in five okra fruits were identified and quantitated in Table 2. The contents of phenolic compounds were significantly different because of different cultivars. One of the phenolic acids, protocatechuic acid, was detected in the five okra fruits. The contents of protocatechuic acid in different okra fruits ranged from 21.59 µg/g DW (“Royal red”) to 116.63 µg/g DW (“Wufu”). In addition, four flavonoids were detected in all these five okra fruits, including quercetin-3-O-gentiobioside, quercetin, rutin, and isoquercitrin. The contents of flavonoids were also significantly different (*p* ≤ 0.05) among the five tested okra fruits. The contents of quercetin-3-O-gentiobioside in different okra fruit ranged from 654.75 µg/g DW to 1703.24 µg/g DW, and the highest content of quercetin-3-O-gentiobioside was detected in “Shuiguo” among all tested samples. The content of rutin ranged from 22.73 µg/g DW to 44.85 µg/g DW, and the highest content of rutin was detected in “Shuiguo” among all tested samples. The content of quercetin ranged from 1.19 µg/g DW to 11.47 µg/g DW, and the highest content of quercetin was detected in “Shuiguo” among all tested samples, which was similar to rutin and quercetin-3-O-gentiobioside. The contents of isoquercitrin ranged from 380.74 µg/g DW to 1076.96 µg/g DW, and the highest content of isoquercitrin was detected in “Kalong 8” among all tested samples.

In short, the values of TFC and individual phenolic compounds significantly varied among the tested okra fruits. The most abundant flavanol of the tested okra fruits was detected as quercetin-3-O-gentiobioside, which was similar to previous studies [2,15,18]. In addition, the highest content of quercetin-3-O-gentiobioside was detected in “Shuiguo” (1703.24 µg/g DW) among all tested samples.

### 2.2. Antioxidant Capacities of Different Cultivars of Okra Fruits

The contributions of okra fruit for health-promoting benefits are partially due to its antioxidant capacities, and previous studies have reported that the phenolics in okra fruit possess significant antioxidant capacities [11,19,20]. Therefore, the antioxidant capacities of the phenolic compounds in the five okra fruits were evaluated and compared. Table 1 summarizes the antioxidant capacities of these different cultivars of okra fruits, including 2,2-diphenyl-1-picrylhydrazyl radical scavenging capacity (DPPH), 2,2’-azino-bis (3-ethylenzthiazoline-6-sulphonic acid) radical scavenging capacity (ABTS), and ferric reducing antioxidant power (FRAP) data. Results showed that the antioxidant capacities ranged from 13.28 µmol TE/g DW (“Royal red”) to 22.87 µmol TE/g DW (“Shuiguo”) in DPPH assay, from 90.65 µmol TE/g DW (“Royal red”) to 173.99 µmol TE/g DW (“Shuiguo”) in ABTS assay, and from 56.92 µmol TE/g DW (“Royal red”) to 211.36 µmol TE/g DW (“Shuiguo”) in FRAP assay. Results were similar to previous studies [4,5]. All the tested okra fruits showed significant antioxidant capacities, but varied by different cultivars, of which “Shuiguo” showed the highest, followed by “Kalong 8”, “Kalong 3”, and “Wufu”, while “Royal red” showed the lowest, regardless of assay method. The different contents of phenolic compounds might preliminarily contribute to these differences of antioxidant capacities among the tested okra fruits [21]. As shown in Table 1, the antioxidant capacities of all the tested okra fruits determined by DPPH, ABTS, and FRAP assays were positively correlated to the TFC. Indeed, “Kalong 3”, “Kalong 8”, and “Shuiguo” showed much better antioxidant capacities than that of others. Results suggested that these five okra fruits, especially “Shuiguo”, could be applied as natural antioxidants for the development of health-promoting products in the fields of functional foods and pharmaceuticals.

### 2.3. Inhibitory Effects on Digestive Enzymes of Different Cultivars of Okra Fruits

**Figure 2:**
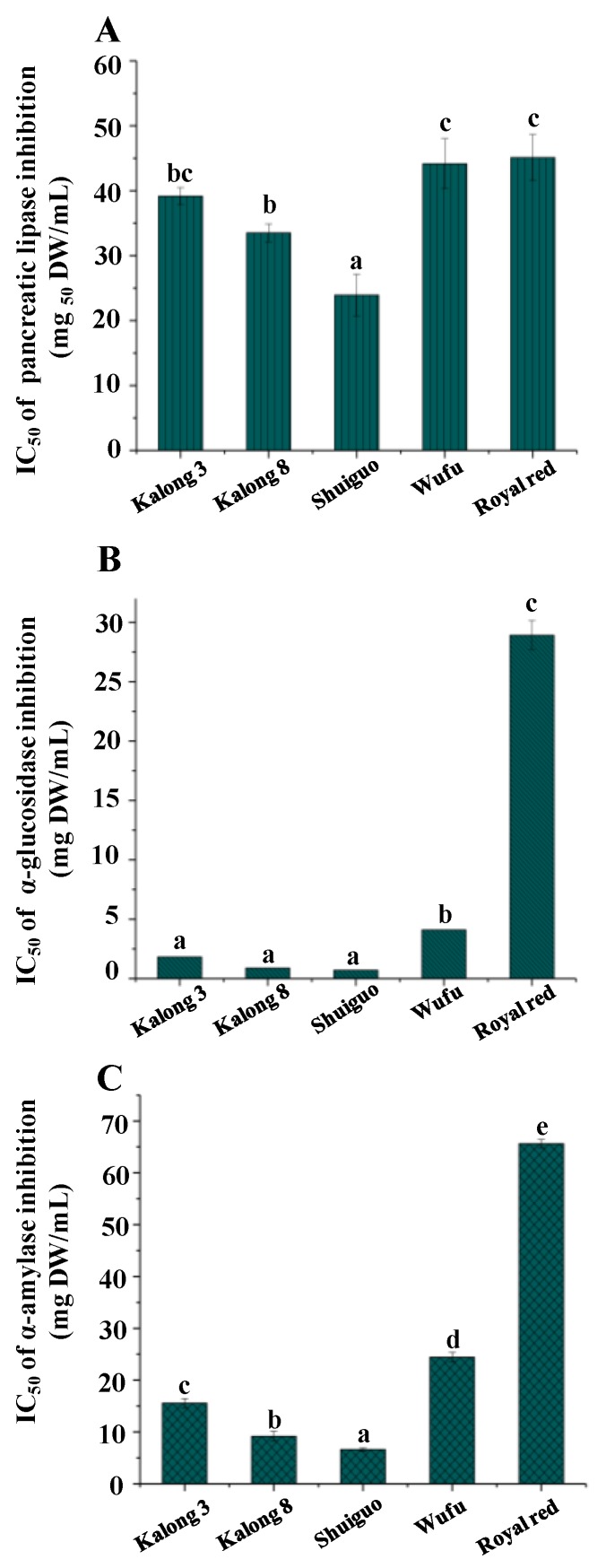
Inhibitory effects on pancreatic lipase (**A**), α-glucosidase (**B**), and α-amylase (**C**) of different cultivars of okra fruits; significant (*p* < 0.05) differences are shown by data bearing different letters (a–e).

One of the keyways to control hyperlipidemia and obesity is to inhibit the pancreatic lipase, thereby inhibiting and delaying the digestion and absorption of triglyceride [22]. Nevertheless, the inhibitory effects on pancreatic lipase of okra fruits from different cultivars have seldom been determined and compared [20]. Figure 2A showed that the inhibitory activities on pancreatic lipase were significantly different (*p* ≤ 0.05) among the tested okra fruits. The IC_50_ values of inhibitory effects on pancreatic lipase varied from 23.92 mg DW/mL to 45.11 mg DW/mL among the tested okra fruits. These five tested okra fruits showed remarkable pancreatic lipase inhibitory activities, of which “Shuiguo” showed the highest among all tested samples, whereas “Royal red” was the lowest. The differences of IC_50_ values may be due to the different values of TFC in okra fruits, which suggested that the higher content of phenolic compounds, the higher inhibition effects on pancreatic lipase it may exhibit [2,23]. Furthermore, compared to the positive control (orlistat, IC_50_ = 6.34 mg/mL), the okra fruit even with the highest level of phenolics presented moderate inhibitory effects on pancreatic lipase (IC_50_ = 23.92 mg DW/mL, “Shuiguo”). Similar results from previously reported studies have shown that quercetin and its derivatives exhibit inhibition effects on pancreatic lipase [24,25], which indicated that the abundant quercetin-3-O-gentiobioside in okra fruit might contribute to the inhibition effects on pancreatic lipase.

The inhibition of α-glucosidase and α-amylase is the main strategies to counteract alterations of metabolism which are associated with the hyperglycemia and type 2 diabetes [26]. Previous studies have reported that the ethanol extract of okra fruits possesses remarkable inhibitory activities on α-glucosidase and α-amylase [7,27]. However, the inhibitory effects on α-glucosidase and α-amylase of different okra fruits have seldom been investigated [20]. As shown in Figure 2B and C, significant differences (*p* < 0.05) were observed among the tested okra fruits in the inhibitory activities on α-glucosidase and α-amylase. The inhibitory effects on α-glucosidase and α-amylase were in an extensive range with the IC_50_ values from 0.71 to 28.91 mg DW/mL, and from 6.59 to 65.60 mg DW/mL, respectively. Particularly, “Shuiguo” exhibited the highest inhibitory activities on both α-glucosidase and α-amylase among all tested samples, which may be associated with its highest value of quercetin-3-O-gentiobioside (Table 2). Previous studies reported that the higher content of quercetin-3-O-gentiobioside in okra fruit might possess higher inhibitory activities on α-glucosidase and α-amylase [7,14]. In addition, compared to the positive control (acarbose, IC_50_ = 4.63 mg/mL), okra fruits, except “Royal red”, which contain high contents of phenolics, exhibited significant inhibitory effects on α-glucosidase. Results suggested that okra fruits could potentially be applied as functional foods for the prevention of hyperglycemia and type 2 diabetes.

### 2.4. Correlations between Phenolic Compounds and Bioactivities

The correlations among phenolic compounds, antioxidant capacities (DPPH, ABTS, and FRAP assays), and inhibitory effects on digestive enzymes (pancreatic lipase, α-glucosidase, and α-amylase) are all summarized in Table 3. Significantly positive correlations were observed between the TFC (r ≥ 0.854) and antioxidant capacities (DPPH, ABTS, and FRAP assays) in Pearson’s correlation analysis. Results suggested that the phenolic compounds might be the main contributors toward the antioxidant capacities of okra fruit, and the higher content of phenolic compounds, the higher antioxidant capacities it might possess in okra fruits, which was similar to previous studies [4,28]. Furthermore, isoquercitrin and quercetin-3-O-gentiobioside were determined to be the main phenolic compounds in okra fruits. Indeed, results revealed that highly positive correlations among individual phenolic compounds and antioxidant capacities, including isoquercitrin (r ≥ 0.878), quercetin (r ≥ 0.888), quercetin-3-O-gentiobioside (r ≥ 0.930), and rutin (r ≥ 0.904).

Furthermore, phenolic compounds were also observed significant correlations with inhibitory effects on digestive enzymes. The TFC (r = −0.617) were observed correlations with the IC_50_ values of pancreatic lipase inhibition, which were similar to previous studies [12,22]. Moreover, results showed that the IC_50_ values for pancreatic lipase was significant (*p* < 0.05) correlated with isoquercitrin (r = −0.825), quercetin-3-O-gentiobioside (r = −0.824), quercetin (r = −0.963), and rutin (r = −0.973). Thus, isoquercitrin and quercetin-3-O-gentiobioside might be mainly contributors to the inhibitory effects on pancreatic lipase in okra fruits because of their high contents (Table 2). Previous studies have also reported similar results that quercetin and its derivatives exhibit inhibitory effects on pancreatic lipase [26,27], suggesting that the abundant quercetin-3-O-gentiobioside in okra fruit may contribute to the inhibitory effects on pancreatic lipase. Moreover, there were also significant (*p* < 0.05) correlations among the inhibitory effects on α-glucosidase and α-amylase with isoquercitrin (r < −0.869) and quercetin-3-O-gentiobioside (r < −0.919), respectively. Actually, previous studies have reported that phenolic compounds, such as quercetin derivatives and proanthocyanidins ((epi)gallocatechins and (epi)catechins) in okra fruits, possess significant inhibitory activities on α-glucosidase and α-amylase [7,13,29]. Thus, because of the high contents of quercetin-3-O-gentiobioside, they may be essential for the inhibitory effects on α-glucosidase and α-amylase in okra fruits. 

## 3. Materials and Methods

### 3.1. Samples and Chemicals

Five representative cultivars of okra fruits, including *A. esculentus* cv. Kalong 3, *A. esculentus* cv. Kalong 8, *A. esculentus* cv. Shuiguo, *A. esculentus* cv. Wufu, and *A. esculentus* cv. Royal red, were harvested from a commercial orchard in Chengdu, Sichuan, China. Six to eight days after anthesis, the fruits with the lengths ranging from 12 to 16 cm were harvested on 12^th^ September 2017. Thirty fruits were harvested, washed with distilled water, frozen and freeze-dried. Finally, the dried samples were screened and stored at −20 °C for further analysis.

Folin–Ciocalteu reagent, gallic acid, vanillin, standard of phenolic compounds (catechin, isoquercitrin, protocatechuic acid, quercetin, quercetin-3-O-gentiobioside, rutin), 6-hydroxy-2,5,7,8-tetramethyl chroman-2-carboxylic acid (Trolox), 2,2-diphenyl-1-picrylhydrazyl (DPPH), 2,2′-azino-bis (3-ethylbenzthiazoline-6-sulphonic acid) (ABTS), pancreatic lipase (4 U/mg), α-glucosidase (10 U/mg), α-amylase (1000 U/mg), *p*-nitrophenyl acetate, *p*-nitrophenyl-α-D-glucopyranoside (*p*NPG), and starch were purchased from Sigma-Aldrich (St. Louis, MO, USA). All reagents and chemicals were of analytical grade.

### 3.2. Extraction of Phenolic Compounds

Phenolic compounds in okra fruits were extracted based on the previous reported method with minor modifications [30]. Firstly, each sample (1.0 g) was extracted with 70% acidified methanol (30 mL, 0.1% HCl, *v*/*v*) in an ultrasonic bath (Kangshijie ultrasound Co., Ltd, Dongguan, Guangzhou, China) (50 kHz, 480 W, ) for 60 min, and centrifugation (Beckman Coulter, Fullerton, CA, USA) at 4000 × g for 10 min at room temperature. The supernatant was collected after centrifugation and the residue was re-extracted again under the same conditions. Two supernatants were mixed and concentrated. Finally, the dried residue was re-dissolved in 10.0 mL of 70% methanol and stored at −20 °C in the dark for further analysis.

### 3.3. Determination of Total Flavonoids

The content of total flavonoids (TFC) in the extract of each okra fruit was determined based on the previous method [30]. TFC was presented as milligram of rutin equivalents per gram of okra fruit dry weight (mg RE/g DW).

### 3.4. Analyses of Individual Phenolic Compounds

The individual phenolic compounds in different okra fruits were performed by HPLC equipped with a ZORBAX Eclipase XDB-C18 column (250 mm × 4.6 mm, 5 µm) at 25 °C and a diode-array detector (DAD) (Agilent Technologies, Santa Clara, CA, USA) according to our previous study [21]. The chromatographic separation was achieved by gradient elution with 0.5% (*v*/*v*) acetic acid solution and acetonitrile. Samples were eluted as follows: 0 min, 5% B; 5 min, 5% B; 50 min, 5–20% B, 70 min, 20–70% B; 72 min, 70–75% B; 72–77 min, 5% B. Six standards, including protocatechuic acid, catechin, quercetin-3-O-gentiobioside, rutin, isoquercitrin, and quercetin, were used for the qualitative and quantitative analysis of individual phenolic compounds. Detection was made at 280 nm for flavan-3-ols and hydroxybenzoic acids and 360 nm for flavanols, respectively. The linear regression data for the six phenolic compounds investigated were summarized in Table 4. The content of individual phenolic compounds was expressed as microgram per gram dry weight (µg/g DW).

### 3.5. Determination of In Vitro Antioxidant Capacities

The DPPH and ABTS radical scavenging capacities, and ferric reducing antioxidant powers (FRAP) of the extract of each okra fruit were determined based on the previous methods [21,30]. Trolox was used as the standard, and the DPPH and ABTS radical scavenging capacities, as well as the FRAP were presented as µmol of Trolox equivalent per gram of okra fruit dry weight (µmol TE/g DW). 

### 3.6. Determination of In Vitro Inhibitory Effects on Digestive Enzymes

The *in vitro* inhibitory effects on digestive enzymes, including pancreatic lipase, α-glucosidase, and α-amylase, were conducted based on the previous methods [20]. Different concentrations of each sample were determined, and a logarithmic regression curve was established to calculated IC50 values (mg of okra fruit dry weight/mL).

### 3.7. Statistical Analysis

Pearson’s correlation coefficients were performed by Origin 2018 software (EA, San Francisco, CA). All experiments were conducted in triplicate, and data were expressed in means ± standard deviations (*n* ≥ 3). Statistical analyses were performed with SPSS 21.0 software (IBM, Armonk, NY, USA), and the differences among mean values were tested by one-way ANOVA, taking a level of *p* < 0.05 as significant to Duncan’s multiple range test.

## 4. Conclusions

Remarkable variations were observed in phenolic compounds, antioxidant capacities, and inhibitory effects on digestive enzymes of different cultivars of okra fruits collected in China. Isoquercitrin and quercetin-3-O-gentiobioside were detected to be the major individual phenolic compounds in okra fruits. The contents of phenolic compounds in “Shuiguo”, “Kalong 3”, and “Kalong 8” were much higher than those of others. Indeed, “Shuiguo” exerted the highest antioxidant capacities and inhibitory effects on digestive enzymes among all tested samples, which might be due to its high content of TFC, especially the high content of quercetin-3-O-gentiobioside. Therefore, okra fruits, especially “Shuiguo”, can be applied as natural antioxidants and natural inhibitors against hyperlipidemia and hyperglycemia in the fields of functional foods and pharmaceuticals.

## Figures and Tables

**Table 1:** Contents of total flavonoids (TFC), 2,2-diphenyl-1-picrylhydrazyl radical scavenging capacity (DPPH), 2,2′-azino-bis (3-ethylenzthiazoline-6-sulphonic acid) radical scavenging capacity (ABTS), and ferric reducing antioxidant power (FRAP) of different cultivars of okra fruits.

Five Cultivars	TFC (mg RE/g DW)	DPPH (µmol TE/g DW)	ABTS (µmol TE/g DW)	FRAP (µmol TE/g DW)
Kalong 3	3.22 ± 0.31 ^ab^	18.86 ± 0.45 ^bc^	134.02 ± 1.23 ^b^	145.80 ± 1.04 ^c^
Kalong 8	3.03 ± 0.33 ^ab^	19.93 ± 0.44 ^b^	137.15 ± 1.68 ^b^	158.12 ± 1.62 ^b^
Shuiguo	3.39 ± 0.09 ^a^	22.87 ± 1.14 ^a^	173.99 ± 0.17 ^a^	211.36 ± 0.65 ^a^
Wufu	2.94 ± 0.09 ^b^	17.25 ± 0.66 ^c^	114.19 ± 1.32 ^c^	94.76 ± 2.47 ^d^
Royal red	1.75 ± 0.09 ^c^	13.28 ± 0.73 ^d^	90.65 ± 2.28 ^d^	56.92 ± 1.73 ^e^

Each value represents the mean ± standard deviation. Different letters in the same column indicate significant differences at *p* < 0.05.

**Table 2:** Contents of individual phenolic compounds in different cultivars of okra fruits.

Peaks	Phenolic Compounds (µg/g DW)	Okra Fruits				
		Kalong 3	Kalong 8	Shuiguo	Wufu	Royal Red
1	Protocatechuic acid	49.31 ± 0.13 ^d^	101.98 ± 0.30 ^b^	62.95 ± 0.23 ^c^	116.63 ± 0.50 ^a^	21.59 ± 0.25 ^e^
2	Catechin	N.D	N.D	N.D	N.D	N.D
3	Quercetin-3-O-gentiobioside	1322.39 ± 0.23 ^c^	1360.42 ± 0.33 ^b^	1703.24 ± 0.44 ^a^	1260.21 ± 0.53 ^d^	654.75 ± 0.53 ^e^
4	Rutin	29.38 ± 0.49 ^c^	40.21 ± 0.38 ^b^	44.85 ± 0.29 ^a^	24.51 ± 0.29 ^d^	22.73 ± 0.40 ^e^
5	Isoquercitrin	869.97 ± 0.32 ^c^	1076.96 ± 0.45 ^a^	1028.62 ± 0.39^b^	643.14 ± 0.40 ^d^	380.74 ± 0.40 ^e^
6	Quercetin	3.56 ± 0.47 ^c^	4.74 ± 0.25 ^b^	11.47 ± 0.28 ^a^	2.71 ± 0.42 ^c^	1.19 ± 0.22 ^d^
	Total flavonoids	2274.61	2584.31	2851.13	2047.20	1081.00

N.D: the compound cannot be detected; each value represents the mean ± standard deviation; Significant (*p* < 0.05) differences are shown by data bearing different letters (a–e); the peaks were the same as in Figure 1.

**Table 3:** Pearson’s correlation coefficients among phenolic compounds, antioxidant capacities, and inhibitory activities on digestive enzymes of different cultivars of okra fruits.

	TFC	PA	QOG	RU	IS	QU	DPPH	ABTS	FRAP	PL	α-Glu	α-Amy
TFC	1											
PA	0.515	1										
QOG	0.962**	0.477	1									
RU	0.670	0.215	0.807	1								
IS	0.863	0.419	0.882*	0.894*	1							
QU	0.671	0.088	0.836	0.886*	0.709	1						
DPPH	0.915*	0.368	0.979**	0.904*	0.930*	0.888*	1					
ABTS	0.856	0.210	0.946*	0.911*	0.878	0.943*	0.984**	1				
FRAP	0.854	0.204	0.930*	0.934*	0.925*	0.907*	0.983**	0.991**	1			
PL	−0.671	−0.091	−0.824	−0.973**	−0.825	−0.963**	−0.912*	−0.951*	−0.950*	1		
α-Glu	−0.977**	−0.657	−0.919*	−0.630	−0.869	−0.558	−0.864	−0.772	−0.783	0.586	1	
α-Amy	−0.976**	−0.580	−0.953*	−0.757	−0.939*	−0.666	−0.932*	−0.858	−0.875	0.714	0.984**	1

**TFC**, content of total flavonoids; **PA**, protocatechuic acid; **QOG**, quercetin-3-O-gentiobioside; **RU**, rutin; **IS**, isoquercitrin; **QU**, quercetin; **DPPH**, DPPH radical scavenging capacity; **ABTS**, ABTS radical scavenging capacity; **FRAP**, ferric reducing antioxidant power; **PL**, inhibitory effect on pancreatic lipase; **α-Glu**, inhibitory effect on α-glucosidase; **α-Amy**, inhibitory effect on α-amylase; Correlation is significant at *p < 0.05, **p < 0.01 level (two-tailed).

**Table 4:** Linear regression data for the six investigated phenolic compounds.

Phenolic Compounds	Regression Equation	Test Range (μg/mL)	*R^2^*	LOD (μg/mL)	LOQ (μg/mL)
protocatechuic acid	Y = 28.665 × X + 0.3124	0.78–12.48	1.0000	0.26	0.78
quercetin-3-O-gentiobioside	Y = 43.945 × X − 310.13	10.00–320.00	0.9989	3.33	10.00
catechin	Y = 16.756 × X − 9.765	2.00–16.00	0.9999	0.70	2.00
rutin	Y = 30.351 × X − 6.271	1.00–8.00	0.9998	0.33	1.00
isoquercetin	Y = 56.55 × X + 68.103	14.08–112.64	0.9998	0.12	0.44
quercetin	Y = 80.507 × X + 9.5089	0.80–6.40	0.9992	0.26	0.80
